# Human Gingival Fibroblast Adhesion and Proliferation on Hydroxyapatite-Coated Zirconia Abutment Surfaces

**DOI:** 10.3390/ma15103625

**Published:** 2022-05-19

**Authors:** Oskar Bunz, Marie-Christine Steegmann, Korbinian Benz, Holger Testrich, Antje Quade, Ella A. Naumova, Wolfgang H. Arnold, Katja Fricke, Andree Piwowarczyk, Thomas Dittmar

**Affiliations:** 1 Department of Prosthodontics, School of Dentistry, Faculty of Health, Witten/Herdecke University, 58455 Witten, Germany; andree.piwowarczyk@uni-wh.de; 2 Independent Researcher, 55116 Mainz, Germany; mcsteegmann@googlemail.com; 3 Department of Oral Surgery and Dental Emergency Care, Faculty of Health, Witten/Herdecke University, 58455 Witten, Germany; korbinian.benz@uni-wh.de; 4 Research Group of Bioactive Surfaces, INP Greifswald e.V., 17489 Greifswald, Germany; holger.testrich@inp-greifswald.de (H.T.); quade@inp-greifswald.de (A.Q.); fricke@nebula-biocides.de (K.F.); 5 Department of Biological and Material Sciences in Dentistry, School of Dentistry, Faculty of Health, Witten/Herdecke University, 58455 Witten, Germany; ella.naumova@uni-wh.de (E.A.N.); wolfgang.arnold@uni-wh.de (W.H.A.); 6 Institute of Immunology, Center for Biomedical Education and Research (ZBAF), Witten/Herdecke University, 58453 Witten, Germany; thomas.dittmar@uni-wh.de

**Keywords:** ceramic implant, atmospheric plasma spraying, abutment, human gingival fibroblasts

## Abstract

Applying antibacterial coatings to dental implant materials seems reasonable but can have negative influences on desired cell adhesion and healing. In this study, zirconia abutment specimens interacting with gingival tissue were used. The aim was to compare the influence of machined or coated zirconia surfaces on the adhesion and proliferation of human gingival fibroblasts (HGF-1). Surface modifications were performed using atmospheric plasma coating with hydroxyapatite, zinc, and copper. Zirconia specimens were divided into four groups: hydroxyapatite, hydroxyapatite with zinc oxide (ZnO), hydroxyapatite with copper (Cu), and an untreated machined surface. After the characterization of the surface conditions, the morphology of adhered HGF-1 was determined by fluorescence staining and subjected to statistical evaluation. The visual analysis of cell morphology by SEM showed flat, polygonal, and largely adherent fibroblast cells in the untreated group, while round to partially flat cells were recorded in the groups with hydroxyapatite, hydroxyapatite + ZnO, and hydroxyapatite + Cu. The cell membranes in the hydroxyapatite + ZnO and hydroxyapatite + Cu groups appeared porous. The results show that HGF-1 adhere and proliferate well on machined zirconia, while plasma coating with hydroxyapatite or hydroxyapatite mixtures does not lead to increased adhesion or proliferation.

## 1. Introduction

A dental implant can be used to replace a tooth, including the root, by screwing an artificial root replacement into the jawbone. Through Branemark et al., implantology has become an integral part of dentistry [1]. With increasing experience and the further development of implant surfaces, the scientific focus has changed from investigating osseointegration to investigating the peri-implant soft tissue.

Accumulations of plaque and bacteria on implant surfaces are known to lead to the inflammation of the peri-implant or surrounding tissue as well as the gingiva, thus contributing to treatment failure [2,3]. In numerous studies, the bacterial plaque biofilm has been shown to be a crucial factor in the development of peri-implant mucositis [4]. It is this plaque biofilm that triggers an initial immune response in the host soft tissue, comparable to that on natural teeth [5].

In peri-implant mucositis, plaque accumulation is the main etiologic factor, and peri-implantitis is also plaque-associated, making it clear that insufficient peri-implant soft tissue facilitates submucosal plaque accumulation or implant/abutment surface contamination [6]. Thus, an immobile keratinized mucosa around the dental implant can protect against bone loss.

If the composition of gingival tissue on a healthy tooth is compared with that on a dental implant, similarities and minor differences become apparent. The differences concern the percentage distribution of the tissue types as well as the morphology and organization of the cells [7], and these differences result from the absence of root cement. Instead of a direct attachment, there is more of an adhesion of the supracrestal connective tissue. These fibers also do not run vertically into the root cementum as in the natural tooth, but mainly run parallel to the abutment surface [7]. Additionally, the gingiva around natural teeth has a higher proportion of blood vessels than the peri-implant soft tissue [8].

Fibroblasts appear to have less contact with the implant surface than with natural teeth. This seems to be limited to fibroblasts only, as collagen fibers, for example, seem to occur normally on implant surfaces [9].

Knowing that the main factor of peri-implant inflammation is bacterial adhesion, many techniques of antibacterial treatment have been developed. For example, there are various techniques for mechanical debridement using ultrasound, air abrasion, or laser or antibiotic therapy [10]. Because these techniques do not always work, there are considerations made to create an antibacterial implant surface. One technique for coating implants is atmospheric plasma spraying (APS), which is the most widely used technique for coating implants among thermal spraying methods [11,12]. Plasma coatings of hydroxyapatite on (metallic) implants have been used in dentistry and orthopedics since the 1980s [13]. To date, this process has mostly been used on metallic substrates, with a few also considering other materials, such as PEEK or composites [14,15]. In most cases, this technique is used to create a more attractive surface for better implant healing. However, it is also possible to apply antibacterial elements on the surface [16].

The purpose of this study was to investigate whether treating zirconia surfaces with an additional coating of hydroxyapatite and antibacterial substances leads to a surface that is attractive to fibroblasts. The surface properties after coating and the influence of machined or coated yttrium-stabilized zirconia surfaces on the adhesion and proliferation of human gingival fibroblasts were analyzed.

## 2. Materials and Methods

### 2.1. CAD/CAM Fabrication of Zirconia Specimens

The specimens were provided by VITA Zahnfabrik (Bad Säckingen, Germany). They were made of yttrium-stabilized zirconia (3-YSZ), which is used as a one-piece, cylindroconical screw ceramic implant (ceramic.implant, vitaclinical; VITA Zahnfabrik, Bad Säckingen, Germany) [17].

The specimens were provided as circular discs with a diameter of 13 mm and a height of 2 mm. They all had a machined surface when supplied. 

### 2.2. Surface Treatment

The modification of the sample surfaces was carried out at the Leibniz Institute for Plasma Science and Technology e.V. (INP, Greifswald, Germany) using plasma spray coating. In this process, ceramics or metals are partially or completely melted by means of a thermal plasma and deposited onto surfaces at speeds of up to 450 m/s. The coating was applied using the plasma spray torch Oerlikon Metco MultiCoat, F4MB-XL (Darmstadt, Germany). The powders used were “Metco 6902” (hydroxyapatite), “Metco 55” (copper) (Oerlikon metco, Kelsterbach, Germany), and zinc oxide (>99% purity) (Carl Roth GmbH + Co. KG, Karlsruhe, Germany). One sample was sprayed with pure hydroxyapatite, while hydroxyapatite was mixed with 3 wt% copper and 3 wt% zinc oxide for the other two sets of samples, respectively, according to a previous publication [18]. To obtain a uniform mixing of the powders, the powder mixture was treated for 2 h in a rotary mixer. The particle size of the powder varied between 0.5 and 180 µm (Table 1). The plasma spray process was carried out at a spray distance of 8.5 cm with a gas mixture of 40 slm argon and an admixture of 5 slm nitrogen. The spraying procedure was repeated until a dense layer was produced (19.75 µm and 36.49 µm were reached (Table 1)).

### 2.3. Surface Characterization

X-ray photoelectron spectroscopy (XPS) measurements were carried out to determine the composition of the coatings (Axis Supra DLD, Kratos, Manchester, UK). For this purpose, line scans with a distance of 0.5 mm were made over the coatings.

The surface roughness was investigated with the optical measuring system Alicona InfiniteFocus and the computer software Alicona IFM 3.2 (Alicona Imagine GmbH, Raaba/Graz, Austria). For this purpose, the test specimens were aligned at an angle of 90° below the objective lens to ensure the most direct possible view of the surface.

Twelve images (each 508.86 × 407.09 μm) of each group were recorded at 20× magnification. Each of the images was computer analyzed at three different measurement areas (100 × 100 μm) with respect to the surface texture Sa (in μm).

The data were checked for normal distribution using the Kolmogorov–Smirnov test (GraphPad Prism, Version 9.0.1, GraphPad Software, La Jolla, CA, USA). Since the data were not normally distributed, significance was determined using the Kruskal–Wallis test and post hoc test via Dunn’s multiple comparisons (alpha level 0.05).

### 2.4. Cell Culture

The specimens were placed into 24-well plates using sterilized forceps, each without touching the surface, and disinfected in 70% ethanol for 20 min. Subsequently, the test specimens were washed three times with phosphate-buffered saline (PBS) (PAN-Biotech GmbH, Aidenbach, Germany) for 5 min each.

Human gingival fibroblasts (HGF-1, LOT 70001246, ATCC, Manassas, VA, USA) were selected for study. HGF-1 cells were cultured in Dulbecco’s Modified Eagle Medium (DMEM) (Sigma-Aldrich, St. Louis, MO, USA) supplemented with 10% Fetal Calf Serum (FCS) (c.c.pro, Oberdorla, Germany), 1% penicillin/streptomycin (PAN-Biotech GmbH, Aidenbach, Germany), and Fibroblast Growth Factor (FGF) (100 µg/mL, Sigma-Aldrich, St. Louis, MO, USA) at 37 °C and 5% CO_2_ in a humidified atmosphere incubator. The medium was changed daily, as this had a positive effect on cell growth. The sufficient proliferation of fibroblasts was evident in passages five to seven, and only these passages were used for the experiments.

To detect HGF-1 fibroblasts on the specimen’s surface, the cells were stained with 5-chloromethylfluorescein diacetate CMFDA (CellTracker^TM^ Green; ThermoFisher Scientific, Waltham, USA). For this purpose, cells were incubated in 5 µM CellTracker^TM^ Green for 45 min at 37 °C and then centrifuged and washed once with PBS (PAN-Biotech GmbH, Aidenbach, Germany). Then, 15,000 cells per specimen were seeded and cultured either for 24 h or for 72 h.

### 2.5. Confocal Laser Scanning Microscopy

To determine the adhesion and proliferation of HGF-1 on the surfaces of specimens, images were obtained byusing a Leica TCS SP5 confocal laser scanning microscope (Leica, Wetzlar, Germany) from all test groups (*n* = 12/group) at time point 1 (after 24-h cultivation) and from all test groups at time point 2 (*n* = 6/group; after 24 h and 72 h of cultivation).

The test specimens were embedded in culture medium on a chamber slide. Sixteen images were taken of each specimen, with the edge length of the captured image being approximately 180 µm. The images/data were analyzed using the ImageJ software (https://imagej.nih.gov/ij/download.html, accessed on 11 February 2022). The mean value was then determined for each test specimen and statistically evaluated. Normal distribution was checked using the Kolmogorov–Smirnov test (GraphPad Prism, Version 9.0.1, GraphPad Software, La Jolla, CA, USA). As the data were normally distributed, one-way ANOVA was used for statistical evaluation as compared to the untreated control, and the significance level was determined to be *p* ≤ 0.05.

### 2.6. Scanning Electron Microscopy

The morphological examination of the surface of the test specimens was performed using a scanning electron microscope (SEM) (Sigma VP, Carl Zeiss AG, Oberkochen, Germany) at two time points. Time point T0 shows the three processed test specimen groups after modification by INP in Greifswald and the control group without HFG-1. Time point T1 shows the four different surfaces after the 24 h cultivation of HGF-1 on the test specimens.

The test specimens with adhered cells had to be fixed in advance. For fixation, 18 mL polyvinylpyrrolidone/sodium nitrite was mixed in sodium cacodylate buffer and 2 mL glutardialdehyde and incubated for 1 h at 4 °C. The test samples were then rinsed with 0.1 M sodium cacodylate buffer (pH 7.3) three times for 10 min each. For glycocalyx preparation, the test specimens were stored in arginine HCl solution at room temperature for 18 h and then rinsed with distilled water three times for 5 min each. Then, the specimens were placed in a solution of tannin/guanidine HCl solution at room temperature for 5.5 h each. This was followed by rinsing for 5 min with distilled water and three times for 5 min each with sodium cacodylate buffer. Osmylation was performed by incubating the test specimens in 1% OsO_4_ in sodium cacodylate buffer for 30 min and then placing them in sodium cacodylate buffer three times for 10 min each. For dehydration or drying, the test specimens remained in 50%, 70%, 90%, and absolute isopropanol for 15 min each and then in 50%, 75%, and 100% acetone (with isopropanol) for 15 min each. After transferring the test specimens to acetone, they were coated with gold–palladium in the SDC 050 sputter coater (BAL-TEC AG Negruet 7, FL-9496, Balzers, Liechtenstein).

Using the computer software SmartSEM (Carl Zeiss AG, Oberkochen, Germany), standardized imaging was performed in a variable low vacuum, with a voltage of 2–10 kV and a magnification of 500×.

## 3. Results

After the coating procedures, the specimen surfaces had varying surface textures which were analyzed before the cell culture experiments took place.

**Figure 1:**
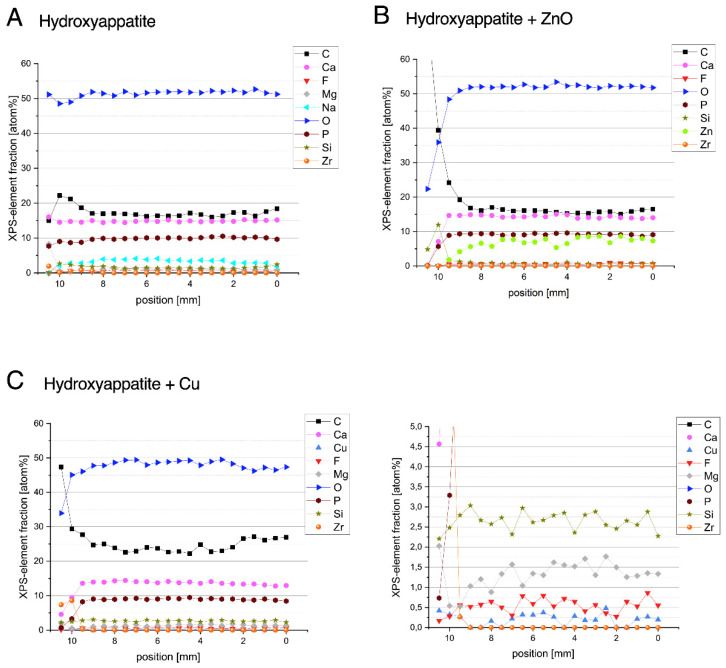
XPS analyses show the chemical composition of specimens after coating with hydroxyapatite (**A**) and hydroxyapatite with zinc (**B**) or copper (**C**). On the right side, an additional extract is shown for better visualization of the amount of copper.

XPS measurements showed the chemical composition of the coated surfaces. In the case of the hydroxyapatite (HAp), a chemically homogeneous layer was measured: ~50 at% O, ~15 at% C, ~15 at% Ca, ~10 at% P, <5 at% Na, and traces of Si, Mg, and F. Zr is visible from the substrate at the edge, which can be attributed to shading by the sample holder (Figure 1A). 

The specimens coated with HAp in combination with 3 wt% ZnO resulted in ~50 at% O, ~15 at% C, ~15 at% Ca, ~10 at% P, and ~9–5 at% Zn, with the Zn content becoming slightly lower towards the left edge. There were also detectable traces of F and Si. Deviations from homogeneity were edge effects caused by the specimen holder during the coating process (Figure 1B).

Coatings with HAp in combination with 3 wt% Cu showed a chemical composition of ~50 at% O, ~25 at% C, ~15 at% Ca, and ~10 at% P. Cu could also be detected, but was measured at only ~0.3 at%; the reason for this is that the Cu was masked by the HAp and the information depth of the XPS analysis is only about 10 nm. Due to the signal-to-noise ratio, it is not possible to say clearly whether this was oxidized or metallic copper. Furthermore, traces of Si, Mg, and F were found (Figure 1C).

**Figure 2:**
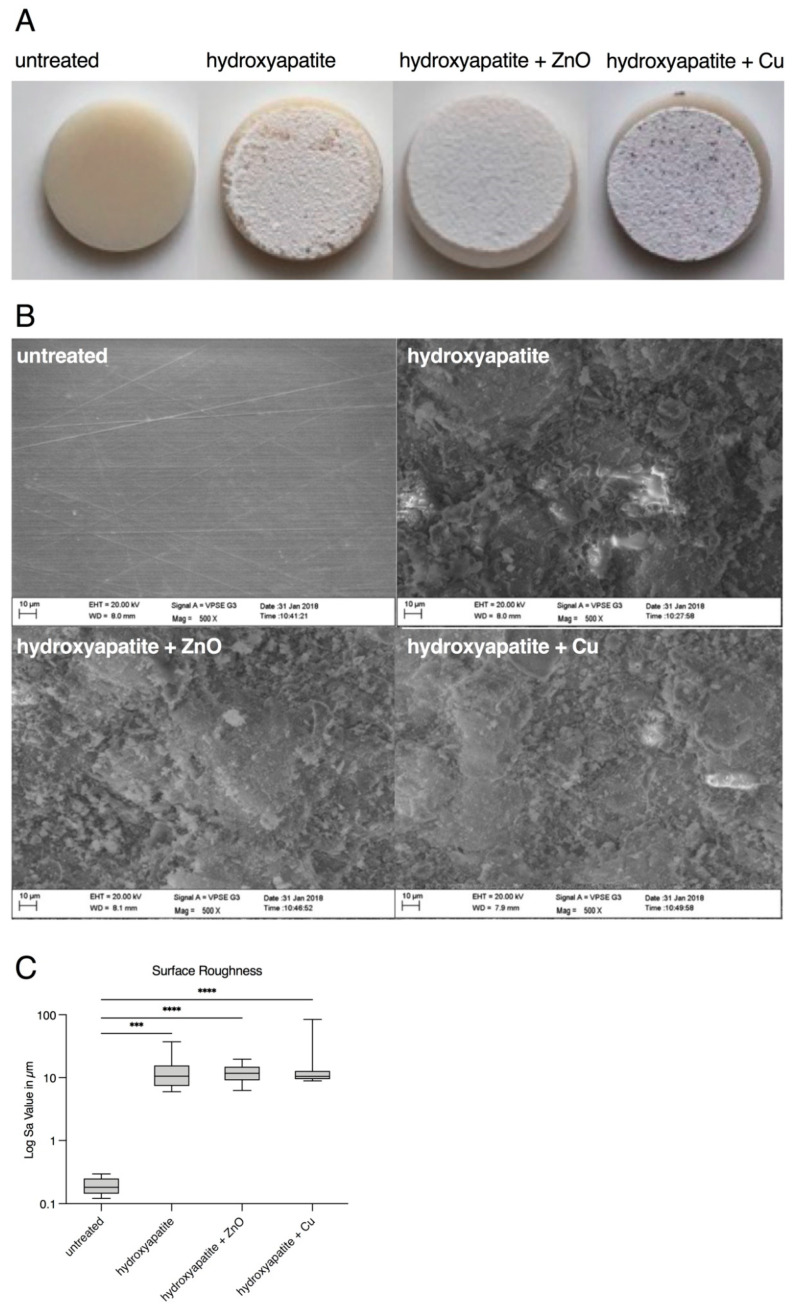
Morphology of specimen surfaces before cell cultivation. (**A**) Photos of specimens. From left to right: untreated, coated with hydroxyapatite, coated with hydroxyapatite + ZnO, and coated with hydroxyapatite + Cu. (**B**) SEM images of specimen surfaces (500× magnification). UL: untreated; UR: coated with hydroxyapatite; LL: coated with hydroxyapatite and zinc oxide (ZnO); LR: coated with hydroxyapatite and copper (Cu). (**C**) Surface roughness (Log Sa values in μm) of untreated and plasma-spayed surfaces. Statistically relevant differences (*p* < 0.05) were found between untreated and treated specimens. *** *p* < 0.0005; **** *p* < 0.00005.

Upon visually examining the uncoated surfaces, they appeared smooth and absent of visible irregularities, whereas the coated specimens were characterized by a white smooth surface (Figure 2A). SEM images at 500× magnification confirmed this observation. The untreated specimens of the control group showed a smooth surface with very fine scratches due to the machining process. In contrast, test specimens with hydroxyapatite coating showed an uneven cloud-like surface (Figure 2B).

The surfaces were also examined quantitatively. The surface texture (measured in Sa) increased significantly for the hydroxyapatite coatings (Figure 2C, Table 2). The median surface texture was 0.1804 μm for the untreated zirconia specimens. The values for pure hydroxyapatite resulted in a median value of 10.55 μm, hydroxyapatite with zinc oxide was 11.75 μm and hydroxyapatite with copper was 10.47 μm.

**Figure 3:**
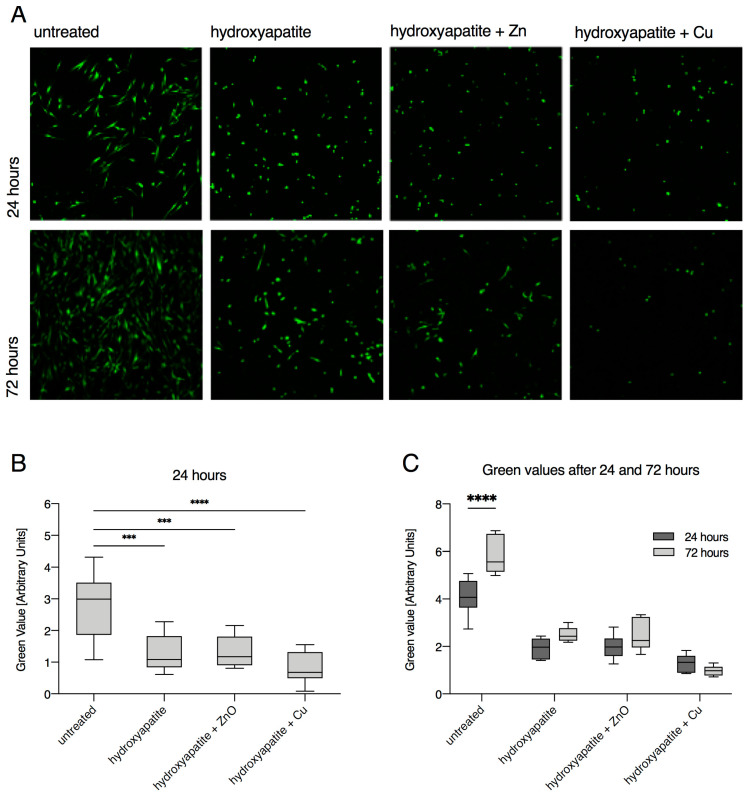
Adhesion and proliferation of human gingival fibroblasts (HGF-1) on uncoated and coated zirconia surfaces. (**A**) Confocal laser scanning microscopy images of specimens. HGF-1 is shown in green (green value) by the fluorescent dye CMFDA. Shown are representative images after 24 h and 72 h of cell cultivation. (**B**) HGF-1 green value after 24 h of cell cultivation. Statistically relevant differences (*p* < 0.05) were found between untreated and treated specimens. *** *p* < 0.0005; **** *p* < 0.00005. (**C**) Upon comparing results for the HGF-1 green value after 24 h and 72 h of cultivation, statistically significant increases (*p* < 0.05) were found for untreated specimens. **** *p* < 0.00005.

After the detailed characterization of the chemical composition and surface properties, the response of human gingival fibroblasts was investigated. Green value determination after 24 h and 72 h did not yield consistent results for all materials. A positive attachment of HGF-1 cells onto uncoated specimens in terms of number and spreading was observed after 24 h (Figure 3A,B and Table 3). Notably, more HGF-1 cells were observed after 72 h, indicating that the adhered cells were viable and able to proliferate (Figure 3A–C, Table 3 and Table 4). In contrast, markedly fewer cells adhered to specimens with hydroxyapatite, hydroxyapatite + ZnO, and hydroxyapatite + Cu (Figure 3A,C and Table 4). However, despite a lower attachment of HGF-1 cells to hydroxyapatite and hydroxyapatite + ZnO, a slightly increased green fluorescence was determined after 72 h, indicating that HGF-1 cells were viable and able to proliferate (Figure 3A,C and Table 4). In contrast, markedly fewer HGF-1 cells were found on hydroxyapatite + Cu specimens after 72 h as compared to 24 h (Figure 3C, Table 4), which might be attributed to the decreased adherence of the cells. Indeed, confocal laser scanning microscopy data revealed a more round-shaped morphology of HGF-1 cells on hydroxyapatite + Cu specimens in comparison to a more elongated and fibroblast morphology of cells on untreated, hydroxyapatite-coated, and hydroxyapatite + ZnO-coated specimens (Figure 3A).

**Figure 4:**
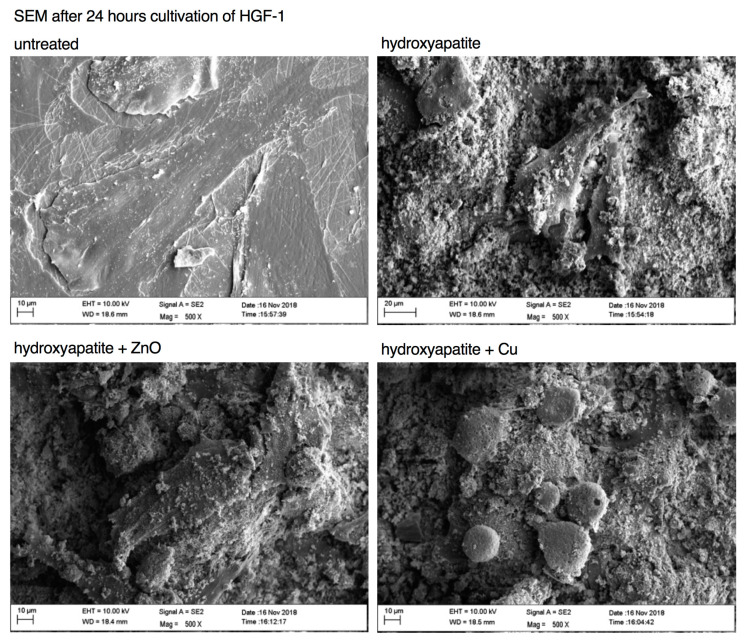
Representative SEM images of the specimens with attached HGF-1 cells at 500× magnification. UL: untreated; UR: coated with hydroxyapatite; LL: coated with hydroxyapatite +ZnO; LR: coated with hydroxyapatite + Cu.

A more in-depth analysis of the morphology of HGF-1 cells seeded onto uncoated and coated specimens was achieved using scanning electron microscopy (Figure 4). Briefly, HGF-1 cells exhibited a typical flat and elongated fibroblast morphology on uncoated specimens. The 500× magnification showed thin, small filamentous secretions, suggestive of proteins and actin filaments (Figure 4).

In contrast, HGF-1 cells on coated surfaces exhibited a markedly different morphology. On surfaces coated with hydroxyapatite and hydroxyapatite +ZnO, HGF-1 cells appeared slightly spread out and had conspicuous deposits on the cell surface, whereas cells on surfaces coated with hydroxyapatite + Cu were markedly smaller and, more importantly, spherical (Figure 4). The SEM data suggested a porous membrane of HGF-1 cells on hydroxyapatite + Cu concomitant with barely visible filamentous protein structures. Briefly, these findings were in line with the confocal laser scanning microscopy data (Figure 3A) and likely indicated a reduced capability of HGF-1 cells to adhere to hydroxyapatite + Cu-coated specimens.

## 4. Discussion

Until now, titanium was considered the most important material for use in dental implants. Its high load-bearing capacity and good compatibility with bone and soft tissue makes titanium an almost perfect material. Although failures do occur, a median 10-year implant survival rate of 94.6% has been reported [19]. One factor leading to failure of dental implants could be the corrosion of titanium, which cannot be prevented in metals [20]. So far, the main alternatives to titanium are made with zirconium dioxide. Some of these are already used as dental implants, but also as abutments. Nevertheless, there is still potential for further developments in preclinic.

For any in vitro model, there are limitations that must be considered. In this model, only one aspect of the complex process between the abutment surface and the biosystem was investigated. The growth and adhesion behavior in association with other mucosal cells, the implant environment, the loading conditions at the tissue-implant interface, the surgical procedure for implant application, and a prolonged healing time were not considered. These aspects can only be studied in a living organism. However, the simplicity of the model used here enables a reproducible environment, a standardized investigation of further cell species from the oral mucosa, and accordingly comparable results. 

In addition to the material itself, the characteristics of the surface play a major role in cellular attachment. Cells are known to behave differently on different surface morphologies, and a rough surface favors bacterial attachment [21,22,23]. On polished surfaces, human gingival fibroblasts (HGF) spread without any particular orientation [24]. On the other hand, fine micro-textures, such as grooves or clusters, may favor the cell configuration, orientation, and proliferation of HGF [24,25,26]. Thus, the surface of these materials must be characterized in addition to the proliferation itself. It is also known that surface roughness up to 0.34 µm Sa does not prevent the attachment of HGF [27]. This could explain the poorer proliferation and adhesion in addition to the effect of hydroxyapatite, zinc oxide, or copper on HGF cells. Nevertheless, slightly increased proliferation was observed in all groups except hydroxyapatite + copper after 72 h of cell cultivation. In our study, roughness values in the range of 10 µm were observed (Figure 2C, Table 2). In plasma coating, surface roughness can be influenced by the choice of powder and spray parameters [28], and thermal post-treatment to reduce roughness is also possible. The modification of the surface texture should be considered in further studies for a possible increase in cell proliferation. Furthermore, it has been shown that an increase in the roughness of implant parts with a connection to soft tissue can also lead to an increase in the inflammation of peri-implant tissue [29]. Although it cannot be excluded that antibacterial substances would be antagonistic, this risk must be taken into consideration when developing new abutments.

In the future, if hydroxyapatite surfaces can be made more attractive to fibroblasts and other cells, it is also reasonable to perform a deeper investigation of adhesion and proliferation. Changes at the DNA and protein level related to roughness have already been shown in osteoblasts [30].

In the present study, antimicrobial essential trace elements of zinc oxide and copper were used. The properties of zinc oxide inhibit the acid production of bacteria in the oral cavity [31], and inhibition has been observed in Gram-negative bacteria [32]. The trace element copper shows antibacterial effects on *S. mutans* and *P. gingivalis* in vitro [33]. However, selective inhibition may result in the greater growth of other species [34]. Abrahamsson et al. showed that the ideal transmucosal surface of an implant should promote the rapid growth of soft tissue cells while reducing bacteria [35]. However, antibacterial substances should not also have a negative influence on the cell growth of fibroblasts. A shown here, HGF-1 cells seeded onto hydroxyapatite + Cu possessed a more spherical morphology, suggesting that the ability of the cells to adhere was hampered, which was further associated with impaired proliferation. Whether these findings indicate that antibacterial compounds such as Cu could have a negative effect on the adhesion and proliferation of gingival fibroblasts remains to be elucidated in further studies.

## 5. Conclusions

Despite the limitations caused by the in vitro model, some findings were obtained by using non-immortalized human fibroblasts (HGF-1) in this study. The results show that HGF-1 cells adhere and proliferate well on machined zirconia, whereas additional plasma coatings with hydroxyapatite or hydroxyapatite mixtures with zinc oxide and copper do not result in increased adhesion or proliferation. Even the machined and untreated zirconia surface appears to be sufficiently attractive to fibroblasts. 

## Figures and Tables

**Table 1:** On the left: Particle sizes of the powder hydroxyapatite (HAp), copper (Cu), and zinc oxide (ZnO) used. The median particle size (d_(p)_) and range are listed in µm. On the right: The coating thicknesses of the surfaces after the coating procedure are shown in µm.

Particle Sizes of the Powder			Coating Thickness	
Powder	d_(p)_ (µm)	Range (µm)	Powder	Thickness (µm)
HAp	93.78	20–180	HAp	36.49
Cu	67.79	20–130	HAp + 3wt% Cu	32.94
ZnO	2.60	0.5–50	HAp + 3 wt% ZnO	19.75

**Table 2:** Descriptive analysis of surface roughness presented in Figure 2C. Median, percentiles, and minimum and maximum Sa values, measured in µm, are shown.

	*n* =	Median	25% Percentile	75% Percentile	Minimum	Maximum
untreated	12	0.18	0.14	0.24	0.12	0.29
hydroxyapatite	12	10.55	7.41	15.61	5.99	37.30
hydroxyapatite + ZnO	12	11.75	9.08	14.95	6.26	19.68
hydroxyapatite + Cu	12	10.47	9.47	12.80	8.88	84.59

**Table 3:** Descriptive analysis (median, percentiles, minimum, and maximum) of HGF-1 green value after 24 h of cell cultivation measured in arbitrary units.

	*n* =	Median	25% Percentile	75% Percentile	Minimum	Maximum
untreated	12	2.99	1.86	3.51	1.076	4.318
hydroxyapatit	12	1.08	0.83	1.82	0.6126	2.277
hydroxyapatite + ZnO	12	1.17	0.90	1.80	0.80	2.15
hydroxyapatite + Cu	12	0.67	0.49	1.31	0.08	1.55

**Table 4:** Descriptive analysis (median, percentiles, minimum, and maximum) of HGF-1 green value after 24 h and 72 h of cell cultivation measured in arbitrary units. Significant differences in the results after 24 h and 72 h are highlighted in grey.

	Cell Cluture	*n* =	Median	25% Percentile	75% Percentile	Minimum	Maximum	Significance *p* ≤ 0.05
untreated	24 h	6	4.06	3.63	4.76	2.73	5.07	<0.0001
	72 h	6	5.56	5.15	6.73	4.99	6.87	
hydroxyapatite	24 h	6	1.97	1.45	2.32	1.40	2.43	0.2841
	72 h	6	2.42	2.24	2.77	2.17	3.01	
hydroxyapatite + ZnO	24 h	6	1.97	1.59	2.33	1.26	2.81	0.4663
	72 h	6	2.24	1.95	3.24	1.66	3.33	
hydroxyapatite + Cu	24 h	6	1.33	0.89	1.60	0.85	1.83	0.7891
	72 h	6	0.97	0.77	1.14	0.71	1.30	

## Data Availability

The data from this study can be shared by the corresponding author upon request.
